# Complete Genome Sequence of Bartonella alsatica Strain IBS 382 (CIP 105477)

**DOI:** 10.1128/MRA.00769-20

**Published:** 2020-08-20

**Authors:** Arno Thibau, Tilman G. Schultze, Wibke Ballhorn, Volkhard A. J. Kempf

**Affiliations:** a University Hospital, Goethe-University, Institute for Medical Microbiology and Infection Control, Frankfurt am Main, Germany; University of Southern California

## Abstract

Bartonella alsatica causes bacteremia in rabbits and, rarely, human infections. Here, we announce the complete and closed genome of B. alsatica IBS 382 (CIP 105477), generated by long-read Pacific Biosciences single-molecule real-time (SMRT) sequencing. The availability of this genome sequence allows future work on understanding the zoonotic potential of this pathogen.

## ANNOUNCEMENT

Bartonella alsatica is a Gram-negative alphaproteobacterium that causes asymptomatic bacteremia in its natural host, the European rabbit (Oryctolagus cuniculus), and is presumably transferred via fleas (Spilopsyllus cuniculi and Xenopsylla cunicularis) (1–4). Since 2006, four cases of human B. alsatica infections have been reported (i.e., endocarditis [5, 6], lymphadenitis [7], and prosthetic vascular graft infection [8]).

B. alsatica strain IBS 382 (CIP 105477) was first isolated from wild rabbits in France (9). Recently, B. alsatica was detected in the desert cottontail rabbit (Sylvilagus audubonii) in the United States (10, 11) and possibly in Thrichomys fosteri rodents in Brazil (hypothesized to be derived from a different evolutionary sublineage) (12). B. alsatica is mostly characterized using the 16S rRNA gene, the 16S-23S rRNA gene intergenic spacer region, or *gltA* and *ftsZ* (5, 7, 9, 12). Previous whole-genome shotgun sequencing approaches using Illumina technology resulted in 21 contigs and an ALLPATHS-assembled scaffold (GenBank accession number JH725020.1). To stimulate future work on understanding the zoonotic potential of this pathogen, we aimed at producing a single-contig sequence for the first isolate of this species.

B. alsatica IBS 382 (CIP 105477) was cultivated in 20 ml *Bartonella* liquid medium (13), incubated for 4 days at 37°C in 5% CO_2_ at 120 rpm, and washed three times with phosphate-buffered saline (pH 7.0 to 7.3) at 6,000 × *g* for 10 min. High-molecular-weight DNA was isolated using the MagAttract HMW DNA kit (Qiagen, Hilden, Germany) and sheared to 12-kb fragments with g-TUBEs (Covaris, Brighton, UK). The sequencing library was prepared following the Pacific Biosciences (PacBio) protocol for SMRTbell libraries using barcoded hairpin adapters (IDT, Leuven, Belgium) and PacBio barcoded adapters for multiplex single-molecule real-time (SMRT) sequencing. Library samples from the same library were sequenced in a single run on a PacBio Sequel instrument utilizing v3.0 sequencing chemistry, Sequel polymerase v3.0, and a SMRT cell v3 LR tray.

Via circular consensus sequencing (CCS), a total of 1,864,869,432 bp were generated, resulting in 14,153 reads with a Phred quality (Q) score above 20 (median score, Q33 [99.95% accuracy]), representing 92,840,004 consensus-corrected bp, and 5,390 reads with a Q score below 20 (median score, Q14 [96% accuracy]), representing 51,701,539 consensus-corrected bp; the average read lengths of the two groups were 6,559 and 9,592 bp, respectively. The raw read *N*_50_ value was 7,814 bp. *De novo* assembly was performed using HGAP v4 (14) with an expected genome size of 2 Mbp, yielding a single contig with a circular sequence of 1,659,117 bp, a GC content of 36.85%, and a mean coverage of 1,503×. The genome was annotated via the NCBI Prokaryotic Genome Annotation Pipeline (PGAP) (15) and rotated to start with *glyA* using SnapGene software (GSL Biotech). All software was run using default parameters.

Various (putative) pathogenicity factors of *Bartonella* spp. (i.e., Bartonella bacilliformis strains KC583 and KC584 and Bartonella henselae strains Marseille URLLY-8 and ATCC 49882 Houston-I) were identified in the genome of B. alsatica (e.g., *Bartonella* adhesin A [BadA] [nucleotide positions 1123087 to 1142468], VirB/D4 locus [nucleotide positions 604101 to 616763], Trw locus [nucleotide positions 832299 to 844919], and Pap31 [nucleotide positions 1257605 to 1258435]). A flagellin apparatus (known in both B. bacilliformis genomes) was not detectable. These observations match the former classification of B. alsatica in *Bartonella* evolution lineage 4 (16).

### Data availability.

The closed genome is accessible via the GenBank accession number CP058235.1 and the associated BioProject and BioSample numbers PRJNA641327 and SAMN15350025, respectively. All long reads for this project were uploaded to the Sequence Read Archive (SRA). The uploaded files include all subreads (accession number SRX8616029), as well as CCS reads filtered by Q scores of Q20 (99% accuracy) (accession number SRX8616028) and Q10 (90% accuracy) (accession number SRX8616027).
